# Enzymatic and environmental degradation of natural rubber–PHBV polymer blends

**DOI:** 10.1007/s10532-025-10241-2

**Published:** 2026-01-08

**Authors:** Carina Frank, Nina Grujicic, Stephanie Strutz, Lhamo Kelsang Yachungtsang, Gregor Trimmel, Lukas Miksch, Lars Gutow, Reinhard Saborowski, Manfred Nachtnebel, Franz Stelzer, Anita Emmerstorfer-Augustin

**Affiliations:** 1 Acib GmbH, Krenngasse 32, 8010 Graz, Austria; 2 Institute for Chemistry and Technology of Materials, Graz University of Technology, NAWI Graz, Stremayrgasse 9, 8010 Graz, Austria; 3 Institute for Molecular Biotechnology, Graz University of Technology, NAWI Graz, Petersgasse 14, 8010 Graz, Austria; 4 Alfred Wegener Institute Helmholtz Centre for Polar and Marine Research, Am Handelshafen 12, 27570 Bremerhaven, Germany; 5 Graz Centre for Electron Microscopy, Steyrergasse 17, 8010 Graz, Austria; 6 BioTechMed-Graz, Mozartgasse, 8010 Graz, Austria

**Keywords:** Polyhydroxyalkanoates, Natural rubber, Depolymerase, Laccase, Biopolymer, Biodegradation

## Abstract

Bio-based and biodegradable polymer blends are promising alternatives to conventional plastics, yet their environmental fate remains poorly understood. Here, we investigated the enzymatic and environmental degradation of natural rubber (NR) blended with poly(3-hydroxybutyrate-co-3-hydroxyvalerate) (PHBV) using recombinant enzymes produced in *Escherichia coli* and *Komagataella phaffii*. Three PHB depolymerases from *Pseudomonas lemoignei* (*Pl*DP), *Acidovorax* sp TP4 (*As*DP), and *Ralstonia pickettii* (*Rp*DP) were isolated and analyzed for thermal and pH stability. All depolymerases efficiently degraded the PHB/PHBV fraction in films and blends, whereas NR was largely resistant to degradation. To enhance rubber degradation, the latex-clearing protein from *Streptomyces* sp. K30 (Lcp_*Ssp*) was produced recombinantly and applied. It showed significant activity on NR substrates, including pretreated PHB–NR blends, demonstrating a synergistic two-step enzymatic process. Scanning electron microscopy and weight-loss assays confirmed selective PHB/PHBV degradation. Separate seawater pH–Stat degradation experiments under environmentally relevant conditions with four hydrolytic enzymes (protease, lipase, esterase, and *Pl*DP), showed highest activity of *Pl*DP on PHB/PHBV–NR blends. Additionally, incubation in estuarine mud revealed progressive surface erosion and pore formation, particularly in PHB-rich blends, highlighting the role of PHB/PHBV in facilitating overall biodegradation. This comprehensive assessment of enzymatic and environmental degradation processes of PHB/PHBV–NR composites provides information to design fully bio-based, degradable polymer materials for sustainable applications.

## Introduction

Bio-based and biodegradable polymers are increasingly investigated as sustainable alternatives to fossil-based plastics. Unlike conventional petroleum-based polymers, bio-based materials are produced from renewable resources such as wood, pulp, fungi, or unicellular organisms. Microbial polymers such as polyhydroxyalkanoates (PHAs) (Pandey et al. 2022; Koller et al. 2025) and polylactic acid (PLA) (Ranakoti et al. 2022) have gained considerable attention because they are both bio-based and biodegradable. Despite these environmental advantages, bio-based plastics often struggle to match the mechanical and processing properties of petrochemical counterparts. A well-known example is polyhydroxybutyrate (PHB), a model PHA that exhibits a high melting temperature as well as pronounced crystallinity and brittleness, which together complicate industrial processing (Marchessault and Yu 2002). Measures to overcome these limitations include the design of copolymers, such as p(3HB-co-4HB) (Jo et al. 2022) or the use of medium-chain-length PHAs (mcl-PHAs, 6–15 carbon atoms per monomer) instead of short-chain-length variants (scl-PHAs, 3–5 carbon atoms per monomer) (Hahn et al. 2024). Both can expand the property range and improve material performance. However, such tailored PHAs remain difficult to obtain at scale, thus limiting their industrial applicability. To overcome these challenges, polymer blending has emerged as an elegant and versatile strategy. The shortcomings of individual components can be mitigated by combining polymers with complementary characteristics. For example, recent studies demonstrated that blending PHB with natural rubber (NR) from the rubber tree *Hevea brasiliensis* results in NR/PHB composites with reduced crystallinity and enhanced ductility and toughness (Bhatt et al. 2008; Zhao et al. 2019; Frank et al. 2023).

Beyond their renewable origin, a central advantage of many bio-based polymers is their biodegradability, a property tightly linked to sustainability and circular economy concepts. In natural settings, biodegradation is often driven by microbial or fungal consortia, whose synergistic enzymatic activities accelerate the breakdown process (Skariyachan et al. 2016; Roberts et al. 2020; Zeghal et al. 2021; Salinas et al. 2023, 2024; Efremenko et al. 2024).

Polyhydroxyalkanoates (PHAs) are naturally synthesized by microorganisms as intracellular carbon and energy storage compounds and can be enzymatically degraded by PHA depolymerases (Jendrossek and Handrick 2002). These enzymes are members of the carboxylic ester hydrolase family (EC 3.1.1). Native depolymerases are further distinguished by their biological function: intracellular enzymes degrade PHA granules accumulated in the cytosol, whereas extracellular enzymes are secreted to hydrolyze PHA in the immediate environment (Jendrossek and Handrick 2002). Intracellularly, PHA granules occur in a 100% amorphous state (Amor et al. 1991), which provides a certain level of flexibility and monomer mobility (de Koning and Lemstra 1992; Jendrossek and Handrick 2002). Extracellular PHA homo-polymers like Poly-(3hydroxy butyrate), P3HB, crystallize with a crystallinity degree of 50–60% (Jendrossek and Handrick 2002), although it usually already contains up to 5% of co-valerate units. Accordingly, the activity of a given depolymerase is strongly influenced by the physical state of the PHA substrate (Koike et al. 2024). Crystallization can be reduced by copolymerization and blending with highly amorphous low Tg polymers like various rubbers, which might be biodegradable, as well, but usually at a much lower rate.

Enzymatic degradation of natural rubber (NR) is exclusively catalyzed by oxidoreductases. They typically exhibit lower catalytic turnover than hydrolytic enzymes due to the challenging nature of oxidative cleavage, which requires an oxidative environment, cofactors, and molecular oxygen. NR is commonly modified by crosslinking prior to industrial use. Biodegradation by bacterial consortia has been reported for NR (Rose and Steinbüchel 2005; Guajardo and Andler 2024), as well as for vulcanized natural rubber (VNR) (Bode et al. 2000; Cheng et al. 2024; Guajardo and Andler 2024), and deprotonated natural rubber (DPNR) (Nguyen et al. 2020). However, the variety of additives introduced during material modification can significantly impair efficient microbial degradation (Rose and Steinbüchel 2005; Chittella et al. 2021).

Composite materials of unmodified NR and PHAs are of superior material characteristics compared to their single material precursors (Bhatt et al. 2008; Frank et al. 2023). Yet, an efficient biodegradation strategy for NR/PHA blends is still pending. In this study, we prepared and isolated three recombinant PHB-depolymerases from soil bacteria and a latex clearing protein (Lcp). We systematically investigated the biodegradability of NR/PHBV blends using combined enzyme treatments at the laboratory scale, as well as natural microbial degradation in estuarine mud. Beyond identifying effective biocatalysts, this study contributes to a deeper understanding of potential degradation pathways of NR/PHBV blends, including enzymatic specificities and synergistic effects that govern polymer disassembly. Finally, we aim to provide insight into the environmental fate of NR/PHBV blends and to support their development as more sustainable alternatives to conventional plastics.

## Materials and methods

### Cloning of recombinant genes

Genes for *As*DP (*Acidovorax* sp TP4 PHB depolymerase, Genbank accession number: BAA35137.1) and *Rp*DP (*Ralstonia picketti* PHB depolymerase, UniProt accession number P12625) were codon-optimized for *Escherichia coli* and ordered from Thermo Fisher Scientific Inc. (St. Leon‐Rot, Germany). A His_6_-tag was fused in-frame to the 5’-end of the genes *As*DP and *Rp*DP. The synthetic genes and the plasmid pK471 containing *Pl*DP (*Pseudomonas lemoignei* PHB depolymerase, UniProt accession number P52090) (Lyshtva et al. 2024) were cut with *Nde*I and *Hind*III and ligated using standard cloning techniques. All constructs were verified by restriction analysis and DNA sequencing, and electroporated into *E. coli* BL21 (DE3) cells for gene expression. The coding sequence for Lcp*_*K30 (*Streptomyces* sp. (strain K30) rubber oxidase, UniProt accession number Q3L8N0) was codon-optimized for *Komagataella phaffii,* ordered from Thermo Fisher Scientific Inc. (St. Leon‐Rot, Germany), and cloned into the vector pPpT4 GAPαS (Näätsaari et al. 2012) with N-terminal His_6_-tag applying Gibson assembly (Gibson et al. 2009). Correct cloning was verified by DNA-sequencing. Electrocompetent *K. phaffii* were prepared and transformed with 500 ng of *Smi*I-linearized vector following the protocol of Lin-Cereghino et al. (2005). The cells were regenerated for 2 h at 28 °C in regeneration medium (YPD/1 M sorbitol 1:1), plated onto YPD_Zeo_ plates, and incubated at 28 °C for 3 days to allow growth of colonies.

### Recombinant protein production

Recombinant *E. coli* BL21 (DE3) cells were cultured overnight at 37 °C and 100 rpm in 500-mL shake flasks containing 50 mL of LB medium supplemented with 30 µg·mL^−1^ kanamycin. Cells were diluted to an OD_600_ of 0.04 in 250 mL of fresh LB medium and grown to an OD_600_ of 0.6–0.8 at 37 °C and 100 rpm. Induction was initiated by adding 0.1 mmol·L^−1^ isopropyl-β-D-thiogalactopyranoside (IPTG), and expression was carried out for 16–18 h at 25 °C.

For gene expression in *K. phaffii*, single colonies were used to inoculate 50 mL of BMGY medium (1% yeast extract, 2% peptone, 100 mmol·L^−1^ potassium phosphate pH 6.0, 1.34% YNB, 4 × 10⁻^5^% biotin, 1% glycerol) and incubated at 28 °C for 48 h. The entire overnight culture (ONC) was used to inoculate 200 mL of BMMY medium (1% yeast extract, 2% peptone, 100 mmol·L^−1^ potassium phosphate pH 6.0, 1.34% YNB, 4 × 10⁻^5^% biotin, 0.5% methanol) to initiate gene expression under the control of the *AOX* promoter. The main culture was incubated at 28 °C for 24 h before 0.05% methanol was added to maintain induction. Methanol supplementation was repeated daily for a total induction period of 48 h. Cells were subsequently harvested by centrifugation at 3,000 g for 5 min, washed twice with deionized water, and either used immediately for cell disruption or stored at − 20 °C.

### Enzyme purification

*Escherichia coli* cell pellets were resuspended in binding buffer (20 mmol·L^−1^ sodium phosphate (NaPi), 20 mmol·L^−1^ imidazole, pH 7.5) and disrupted by sonication (Branson Sonifier 250, 2-s pulse, 4-s pause, 60% amplitude for 6 min). The cell lysate was obtained by centrifugation at 3,000 g for 45 min at 4 °C. The clear supernatant (soluble fraction) was filtered through a 0.45-µm syringe filter and used for subsequent purification. For His-tag affinity purification of the depolymerases, a 5-mL His-Trap column (GE Healthcare) was employed using an ÄktaPrime plus system (GE Healthcare). The protein was bound to the column at a flow rate of 0.5 mL·min^−1^. After washing off unbound proteins, the depolymerase was eluted with a 50 mL gradient of 0 to 500 mmol·L^−1^ imidazole at a flow rate of 2 mL·min^−1^. Ultrafiltration with a 10 kDa VivaSpin® tube (Sartorius) was performed to remove the imidazole and to concentrate the protein, with buffer exchange to 10 mmol·L^−1^ potassium phosphate (KPi), 100 mmol·L^−1^ NaCl, pH 7.9. Protein concentration was determined using the Bradford assay (Bradford 1976), with BSA as the standard for calibration curves. The purified protein was then aliquoted, shock-frozen in liquid nitrogen, and stored at − 20 °C.

*Komagataella phaffii* cell pellets harboring Lcp were resuspended in 50 mmol·L^−1^ sodium acetate buffer (pH 6.5) at a ratio of 1 g of cell wet weight (CWW) per mL of buffer. To inhibit protease activity, 0.1 mmol·L^−1^ phenylmethylsulfonyl fluoride (PMSF) was added. The resuspended cells were then disrupted using a Merckenschlager homogenizer (Braun, Melsungen, Germany) under CO₂ cooling. The cell lysate was centrifuged for 10 min at 3000 g, and the supernatant was collected for further purification steps.

### SDS-PAGE and immunoblot analysis

Four OD₆₀₀ units of cells were collected by brief centrifugation and stored at − 80 °C. Cell pellets were thawed on ice and lysed in 300 µL of lysis buffer containing 1.85 mol·L^−1^ NaOH and 7.5% β-mercaptoethanol. Proteins were precipitated by adding 300 µL of ice-cold 50% trichloroacetic acid (TCA) and incubated on ice for 1 h. The protein precipitates were collected by centrifugation and washed twice with ice-cold water. Resulting protein pellets were dissolved in 50 µL of NuPAGE™ sample buffer (Thermo Fisher Scientific) supplemented with 4% β-mercaptoethanol and 30% 1 mol·L^−1^ Tris, followed by heating at 75 °C for 15 min.

For SDS-PAGE analysis, 10 µL of each sample were separated on 10% Bolt Bis–Tris Plus gels (Thermo Fisher Scientific) and visualized by Coomassie Blue staining. For immunoblot analysis, proteins were electrophoretically transferred onto a nitrocellulose membrane, which was blocked for 1 h with TBST (30.3 g·L⁻^1^ Tris, 87.6 g·L⁻^1^ NaCl, 0.003% Tween 20) containing 5% bovine serum albumin (BSA). Membranes were washed three times for 10 min each with TBST and incubated overnight at 4 °C with a His-tag primary antibody (1:4000; Sigma Aldrich, Vienna, Austria). After washing three times with at least 10 mL TBST, membranes were incubated with an HRP-conjugated secondary antibody (goat anti-rabbit IgG, 1:10,000; Sigma Aldrich) and washed three times with TBST. Immunoreactive bands were visualized using Clarity Max Western ECL Substrate (Bio-Rad, Vienna, Austria). PageRuler™ pre-stained protein ladder (Thermo Fisher Scientific) was used as a molecular weight marker.

### Biopolymers and blend preparation

Standard natural rubber (NR) was provided by Semperit Technische Produkte GmbH (Vienna, Austria). Two different types of poly(3-hydroxybutyrate-co-3-hydroxyvalerate) (PHBV) (PHi001 with approximately 5 wt.% hydroxyvalerate (HV) units and a comparably high amount (> 40 wt.%) of PBAT (poly(butylene adipate-*co*-terephthalate)) as an impact modifier, and PHi002 with approximately 5 wt.% hydroxyvalerate units, without PBAT) were provided by NaturePlast (Ifs, France). Rubber was used as received. The PHBV pellets were dried for 4 h at 60 °C to remove moisture.

A casting method was employed to produce thin PHi001 and PHi002 films. PHi001 and PHi002 pellets were weighed, dissolved in chloroform, and the polymer solutions were magnetically stirred for 24 h at room temperature. The solutions were then cast into Petri dishes and allowed to evaporate in a fume hood for 48 h at room temperature. Dried cast films were ~ 1 cm in diameter, ~ 1 mm in height, and weighed ~ 0.15 g.

Preparation of the polymer blends used in this study has been described previously (Frank et al. 2023). Briefly, PHi001 and PHi002 from NaturePlast were blended with natural rubber (NR) in an internal mixer Plasti-Corder (Brabender, Germany) at 160 °C for 5 min and 70 rpm. Blends with mass ratios of 80/20, 50/50, and 20/80 (NR/PHBV) were prepared, along with the neat polymers as references.

### Enzymatic hydrolysis of biopolymers

Enzyme activity was evaluated with respect to both temperature and pH stability using *p*-nitrophenyl butyrate (pNPB 0.4 mM; Sigma-Aldrich) as a model substrate (Shivakumar 2013, Shivakumar et al. 2011). For thermal stability, purified enzymes were diluted in 10 mmol·L^−1^ potassium phosphate (KPi) buffer (pH 7.5) to a final concentration of 0.5 mg·mL^−1^ and incubated at five different temperatures (30, 37, 45, 50, and 60 °C) for 0, 15, 30, 60, and 120 min. For pH stability, enzymes were pre-incubated at 30 °C for 5 h in different buffers (without substrate) spanning the desired pH range. In both cases, residual activity was determined by measuring the hydrolysis of pNPB. Hydrolysis assay was conducted in 96-well microtiter plates in a total volume of 200 µL. A reaction solution was prepared by diluting the pNPB stock (0.4 mM) 1:100 in 10 mM KPi buffer pH 7.5. Finally, 160 µL of the reaction solution (c_reaction_ = 3.2 µM pNPB) and 40 µL of the treated enzymes (c_reaction_ = 0.1 mg) were mixed directly in the microtiter plate. Absorption at 405 nm was measured after 10 min of incubation with the SynergyMx plate reader (Biotek).

### Assay of Lcp activity

The activity of the latex-clearing protein from *Streptomyces* sp. K30 (Lcp_*Ssp*) was quantified by monitoring the consumption of molecular oxygen in a reaction containing 0.6 mg of enzyme, cis-1,4-polyisoprene latex as substrate (final concentration 0.5%), and 100 mmol·L^−1^ potassium phosphate buffer (pH 7.0) in a total volume of 3 mL. Oxygen consumption was measured at 30 °C using a PyroScience oxygen electrode (Aachen, Germany) with continuous stirring at 300 rpm. Triplicate reactions and controls without enzyme were included. To enhance enzymatic activity and improve substrate accessibility, the polymer blend was pre-treated with PHB depolymerase (*Pl*DP) prior to Lcp-*Ssp* degradation. Pre-treated material was incubated under *Pl*DP optimized conditions (0.1 mg *Pl*DP, 10 mM KPi pH 8.5, 30 °C, 100 rpm), just as described for PHi001 and PHi002 films degradation by *Pl*DP. This pre-treatment modifies the surface of the blend, facilitating more efficient docking and subsequent degradation by Lcp-*Ssp*. The specific activity was calculated as previously described (Andler et al. 2022).

### Weight loss of incubated solvent cast films

The degradation of PHi001 and PHi002 films by three different PHB depolymerases was compared. PHBV films obtained from NaturePlast (Ifs, France) were weighted individually prior to addition of the reaction mixture. Reactions (2 mL final volume) contained PHBV films in 10 mmol·L⁻^1^ potassium phosphate (KPi) buffer adjusted to the optimal pH for each enzyme (*Pl*DP, pH 8.5; *Rp*DP, pH 8.0; *As*DP, pH 8.5). Degradation was initiated by adding 0.1 mg of enzyme, and vials were incubated at 30 °C with gentle shaking (100 rpm) for up to 63 days. At defined time points (1, 5, 13, 20, and 33 days), samples were centrifuged, and the supernatants were carefully decanted without disturbing the residual film material. The remaining solids were dried overnight at 65 °C in an incubator (Sartorius), and the vials were weighed to determine the residual film mass. Afterwards, the remaining material was overlaid again with 1 mL of fresh 10 mmol·L⁻^1^ KPi buffer at the respective optimal pH, and 0.1 mg of fresh enzyme was added. Film degradation was quantified as the percentage of weight loss relative to the initial dry film mass over time.

### Latex glove degradation

A powder-free Sempercare latex glove (Sempermed, Edition LOF) was cut into approximately 1 × 1 cm pieces and incubated with 1 mL of freshly purified Lcp-*Ssp* enzyme to assess enzymatic degradation. Incubations were conducted at 28 °C with an initial enzyme concentration of 1 mg·mL^−1^. As a negative control, latex pieces from the same glove were incubated under identical conditions with buffer alone. To maintain enzyme activity and ensure comparable conditions for the control, the enzyme solution or buffer was replenished every 2 to 3 days. After a total incubation period of 1 month, surface textures were monitored by scanning electron microscopy.

### Scanning Electron Microscopy (SEM)

The surface texture of the various blends before and after enzymatic degradation was observed by scanning electron microscopy (SEM). Samples were directly investigated after the blend preparation. As all samples were electrically non-conductive, classical high vacuum SEM was not applicable without adding a conductive layer. To overcome this issue, an environmental SEM (ESEM) was used (FEI ESEM Quanta 450 FEG, Hillsboro-US). This microscope enables the direct investigation of the polymers by using the so-called “low vacuum mode” (Nachtnebel et al. 2017). To prevent an alteration of the polymer due to interaction with the electron beam (Zankel et al. 2008), a low acceleration voltage of 7.0 kV and a moderate beam current were used. SEM analyses were performed on NR/PHi002 50:50 and treated with 0.1 mg of *Pl*DP. As control samples, untreated NR/PHi002 50:50 blends were studied. The change in the surface morphology of PHBV/NR blends was analyzed after 60 days of enzymatic degradation. Images of each sample were taken at 3,000 × magnification showing the BSE (backscatter electron) contrast.

### Mud exposure

Natural estuarine mud for degradation experiments was collected at low tide from an intertidal sampling area at the Weser estuary off Bremerhaven (53°32′21.3″ N 8°34′33.2″ E) in April 2021. Sediment of the upper 20 cm was taken with a spade and transferred into 20-L buckets. For the transport to the lab, the mud was covered with a thin layer of estuarine water to prevent drying.

Material samples of approximately 1 cm^2^ were cut out from untreated PHi002/NR blends with different NR/PHBV ratios and incubated in the untreated mud. Water, round glass jars (0.5L volume, 7 cm diameter, 10 cm height) and any tool used in preparation of this experiment were autoclaved beforehand at 121 °C for 40 min. Approximately 350 g of the collected mud was placed in each of three glass jars and autoclaved water was added to form a thin layer of 1 cm on top. Three samples of each blend were added to each glass jar and buried approximately 5 cm into the mud, spaced approximately 4 cm to each other. One glass jar with three plastic samples was examined each month resulting in incubation periods of one, two and three months, respectively.

After incubation, the PHi002/NR blends were removed, carefully rinsed with deionized water, dried, and stored separately in sealed freezer bags. Samples were dried at 40 °C for 24 h. Afterwards the samples were glued on SEM-stubs with conductive carbon pads (Plano GmbH Wetzlar, Germany) and sputter-coated with gold/palladium. SEM images of the surfaces were taken with a FEI Quanta 200 device.

### pH–Stat titration

The rate of enzymatic hydrolysis of the NR/Phi002 blends in seawater was measured by pH–Stat titration using a TitroLine® 7000 titrator (SI Analytics GmbH, Mainz, Germany). The titration unit was equipped with a 20 mL exchangeable head and a 1 mm diameter PTFE tube as a titration tip. The unit was connected to a magnetic stirrer (TM 235), a pH-electrode model A 162 2 M DIN ID, and a circulation thermostat (Lauda-Königshofen, Germany). The reaction vial was a 20 mL glass vial placed in a custom-made thermostat jacket to maintain a constant temperature (Miksch et al. 2022).

pH–Stat titration was performed after Miksch et al. (2022) whilst the rate of acidification due to the hydrolysis of the PHi002/NR blends was determined by counter-titration of a base. Prior to use, PHi002/NR blends were ground in a cryogenic mill (SPEX SamplePrep, 6775 Freezer/Mill). The grinding program was set on the following parameters: Pre-cooling time: 15 min; Cycles: 4–8; Grind: 2–1 min; Cool: 2–1 min, Speed: 15 cps. The ground material was sieved for the fraction < 200 µm. The microparticles of the PHi002/NR blends were then suspended in artificial seawater prepared with 3.2% sea salt (Seequasal, Münster, Germany) in deionized water. The suspensions were first stirred in a glass beaker with a magnetic stirring bar at 800 rpm for 16 h before aliquots of 10 mL were subjected to pH–Stat titration. Ten to 100 µL of enzyme solution of commercial lipase from *Candida antarctica* (Sigma P62288), protease from Bacillus licheniformis (Sigma P4860), and esterase from *Bacillus subtilis* (Sigma, P96667), as well as PHB depolymerase (*Pl*DP) were added to the reaction vial with a 100 µL micro-syringe (Model 710 N, Hamilton Bonaduz AG, Bonaduz, Switzerland). The pH was kept constant at 8.2 by titration of 10 mmol·L^−1^ NaOH solution. The supply of NaOH solution after enzyme addition was recorded every minute for 60 min. Each measurement started with an initial recording of the consumption of NaOH solution for 60 min without enzyme, to correct for the effects of atmospheric CO_2_ and polymer autolysis. For each replicate, an additional enzyme blank was measured, where the hydrolysis rate of the enzyme in seawater without substrate was determined, to correct for the autocatalytic activity of the enzyme. The hydrolysis of the three different PHi002/NR blends was assayed at 30 °C. The electrode was calibrated each day before use. Routine measurements were carried out in triplicate.

## Results

### Activity and stability characteristics of PHB depolymerases

**Fig. 1:**
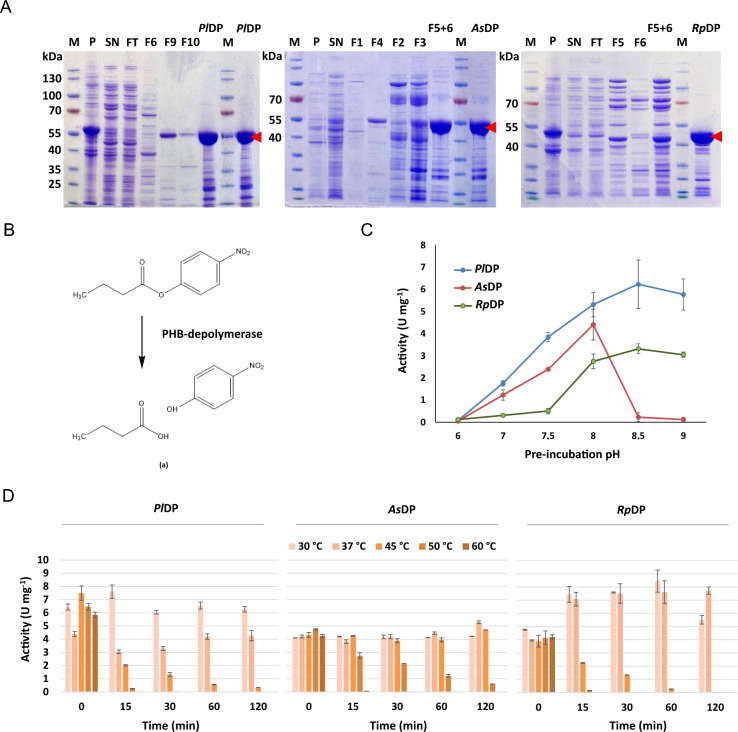
Purification and characterization of *Pl*DP, *As*DP, and *Rp*DP. **A** SDS-PAGE analysis of *Pl*DP, *As*DP, and *Rp*DP purification. FT: flowthrough; P: pellet; SN: supernatant; F1–F10: fractions collected during purification; M: PageRuler™ prestained protein ladder. Red arrows indicate the purified protein bands corresponding to *Pl*DP (55 kDa), *As*DP (55 kDa), and *Rp*DP (42 kDa). **B** Schematic representation of the enzymatic hydrolysis of p-nitrophenyl butyrate (pNPB) by depolymerases, yielding p-nitrophenol (pNP) and butyric acid. **C** pH stability was assessed by pre-incubating the enzyme in 100 mmol·L^−1^ potassium phosphate buffer (pH 6.0–8.0), and 100 mmol·L^−1^ Tris–HCl buffer (pH 8.5 – 9.0), followed by pNPB hydrolysis assay. Assays were conducted in 10 mM KPi buffer, with 0.1 mg enzyme and 3.2 µmol·L^−1^ pNPB at 30 °C. Hydrolysis was measured after 10 min of incubation (means ± SD, n = 3). **D** Thermal stability was assessed by pre-incubating the enzymes at 30–60 °C for different time periods, followed by activity measurement at 30 °C as described for pH stability determination in panel (**C**) (means ± SD, n = 3)

*Pl*DP, *As*DP, and *Rp*DP were heterologously produced in *E. coli* and purified to homogeneity (Fig. 1A). The apparent molecular masses of *Pl*DP and *As*DP were approximately 55 kDa, while *Rp*DP displayed a lower molecular mass of 42 kDa. Enzymatic activity was evaluated using p-nitrophenyl butyrate (pNPB) as a soluble model substrate (Fig. 1B). The three PHB depolymerases were stable at slightly alkaline conditions (Fig. 1C). *Rp*DP showed maximal stability at pH 8.0 (Fig. 1C), whereas *Pl*DP and *As*DP were most stable when incubated at pH 8.5, with decreasing activity under more acidic conditions. Thermal stability assays revealed distinct temperature sensitivities among the depolymerases. Temperatures above 45 °C were detrimental to both *Pl*DP and *Rp*DP (Fig. 1D), while *As*DP remained largely unaffected. *Pl*DP and *Rp*DP were completely inactivated within 15 min at 50 °C and 60 °C and exhibited substantial activity loss at 45 °C after 1 h. This rapid decline in activity at higher temperatures was accompanied by visible precipitation of the respective enzymes. Since all three depolymerases retained stable activity at 30 °C, this temperature was chosen for all subsequent experiments to enable direct comparison across variants.

### PHA depolymerase activities on commercial PHA-materials

**Fig. 2:**
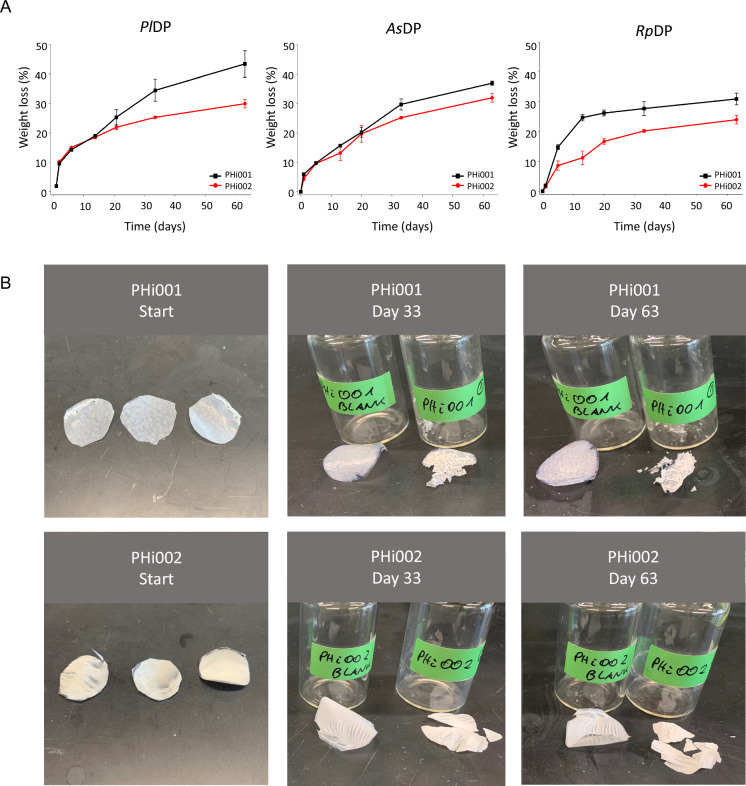
Enzymatic degradation of commercial PHA films by PHB depolymerases. **A** Weight loss of solvent-cast PHi001 and PHi002 films over 63 days of incubation with 0.1 mg *Pl*DP, *As*DP, or *Rp*DP in 2 ml (10 mM KPi) and at 30 °C. Reaction solution pH was adjusted to the optimum of the respective enzyme. (means ± SD, n = 3). **B** Representative images showing the degradation of PHi001 and PHi002 films after 0, 33, and 63 days of incubation with *Pl*DP

*Pl*DP, which also exhibited the highest specific activity on the surrogate substrate pNPB (Fig. 1D), showed the strongest degradation on PHi001 and PHi002 films (Fig. 2). At early stages of degradation, significant weight loss was already observed with *Pl*DP and *As*DP (Fig. 2A). After one day, PHi001 and PHi002 films lost ~ 10% and ~ 5% of their initial weight, respectively. In contrast, *Rp*DP displayed a delayed onset of activity on PHi002, followed by accelerated degradation and a plateau after 13 days, reaching a final weight loss of 31%. For PHi002, all three enzymes induced relatively steady degradation over time, although rates slowed toward the end of the experiment. Across all conditions, PHi001 consistently degraded faster than PHi002.

After 33 days of incubation, PHi002 films had fragmented into pieces (Fig. 2B). The degradation pattern suggested preferential hydrolysis of the PBAT fraction in PHi001, while the PHBV component remained initially resistant. By the end of the 63-day incubation, all films had undergone extensive fragmentation, surface erosion, and progressive weight loss exceeding 24% (Fig. 2A). The strongest effect was observed for PHi001 with *Pl*DP, which reached 43% weight loss and disintegrated into small fragments, whereas PHi002 predominantly broke into larger pieces.

Even though PHi001 was more accessible to enzymatic degradation, we decided to not further focus on it, as PBAT is a petrochemically derived copolyester and therefore not fully biobased. To concentrate on a fully bio-based and biodegradable material, we proceeded with NR/PHi002 for subsequent degradation experiments.

### Degradation of NR/PHB-rubber blends by PHB-depolymerases

**Fig. 3:**
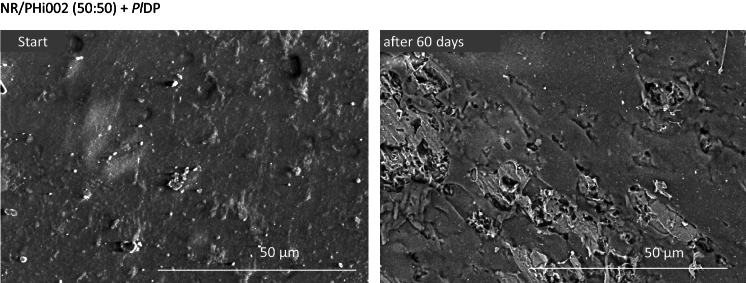
SEM micrographs of NR/PHi002 50:50 blends before and after 60 days of incubation under *Pl*DP optimized conditions (0.1 mg *Pl*DP, 10 mM KPi pH 8.5, 30 °C, 100 rpm). White scale bar: 50 µm

The NR/PHi002 50:50 films exhibited a smooth surface with no clearly visible phase separation, indicating even dispersion of PHBV within the NR matrix (Fig. 3). After enzymatic treatment with *Pl*DP, the surfaces became noticeably roughened, showing pore formation, cracks, holes, and cavities, which are characteristic of targeted surface degradation.

### Enzymatic degradation of natural rubber and NR/PHi002 blends

**Fig. 4:**
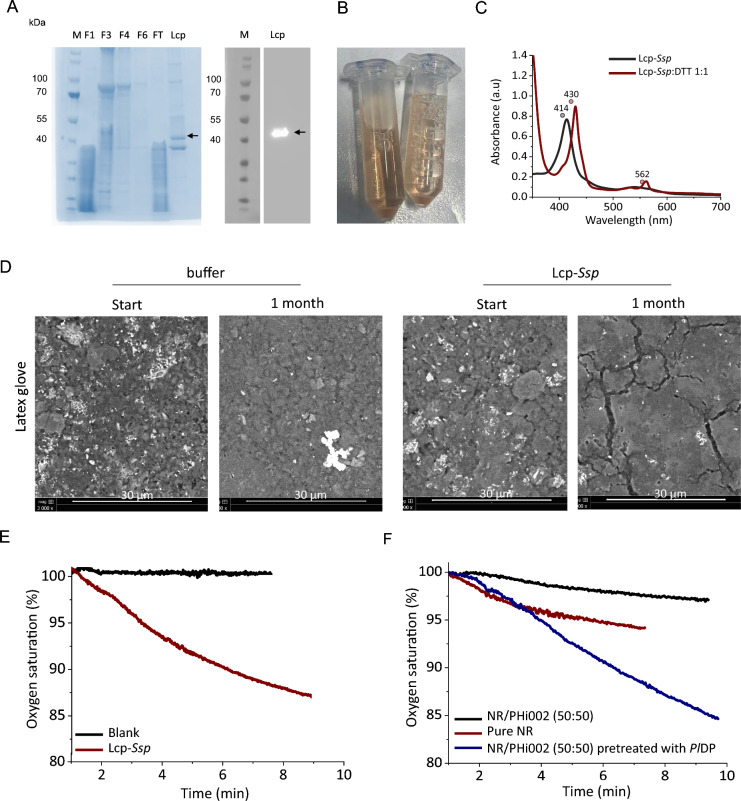
Characterization and enzymatic activity of recombinant Lcp-*Ssp* on natural rubber. **A** SDS-PAGE analysis of Ni–NTA affinity purification fractions of Lcp-*Ssp* expressed in *K. phaffii*. Marker protein (M), fractions 1, 3, 4, 6, flow-through (FT), and purified Lcp-*Ssp* are shown. Immunoblot using Anti-His antibodies confirms Lcp-*Ssp* expression. **B** Purified Lcp-*Ssp* exhibiting the characteristic red-brown color of heme-containing proteins. **C** UV–Vis spectra of purified Lcp-*Ssp* (black line, as-isolated) and dithionite-reduced Lcp-*Ssp* (red line). The reduced spectrum was recorded immediately after addition of 2–5 mg sodium dithionite. **D** SEM micrographs of rubber glove pieces before and after 1 month of enzymatic treatment with 1 mg Lcp-*S*sp at 28 °C. Enzyme solution was renewed every 2–3 days. White scale bar: 30 µm. **E** Oxygen consumption assay showing oxidative cleavage of cis-1,4-polyisoprene latex by 0.6 mg Lcp-*Ssp* in 100 mM KPi pH 7 (V_tot_ = 3 ml), at 30 °C and continuous stirring at 300 rpm. Negative controls without enzyme confirmed that oxygen consumption was enzyme dependent. **F** For comparison, the enzymatic activity of Lcp-*Ssp* on pure NR and NR/PHi002 blends was determined under the same conditions as described for panel (**E**). Pretreatment of the NR/PHi002 (50:50) sample was carried out under *Pl*DP optimized conditions (0.1 mg *Pl*DP, 10 mM KPi pH 8.5, 30 °C, 300 rpm, 168 h) to enhance accessibility

Purified Lcp_*Ssp* was obtained as a single band of approximately 43 kDa, and immunoblot analysis confirmed its identity (Fig. 4A). The protein displayed the characteristic red-brown coloration of heme-containing enzymes (Fig. 4B). UV–visible absorption spectra of the as-isolated and dithionite-reduced forms showed maxima at 414 nm and 438 nm, respectively, consistent with cytochrome-type heme proteins. The redox-dependent shift of the α- and Q-bands further verified proper heme incorporation and folding (Fig. 4C).

To assess enzymatic activity on natural substrates, rubber glove pieces were incubated with Lcp_*Ssp* and examined by scanning electron microscopy (Fig. 4D). Buffer-treated controls remained largely intact, while enzyme-treated samples exhibited pronounced cracks and pores, indicating surface degradation of the polymer matrix. Oxygen consumption assays using *cis*-1,4-polyisoprene latex (Fig. 4E) confirmed a specific activity of 0.68 U·mg^−1^.

The degradation of NR/PHi002 composite materials showed that both untreated and *Pl*DP-pretreated blends were subjected to Lcp_*Ssp*-catalyzed oxidation (Fig. 4E, F). Oxygen consumption was observed in all samples, demonstrating enzymatic activity on both material types. However, *Pl*DP-pretreated blends displayed markedly faster and higher oxygen consumption, indicating that prior removal of PHBV by *Pl*DP enhances accessibility of the rubber phase for Lcp_*Ssp*-mediated degradation.

### NR-PHBV blends are partially degraded under natural environmental conditions

**Fig. 5:**
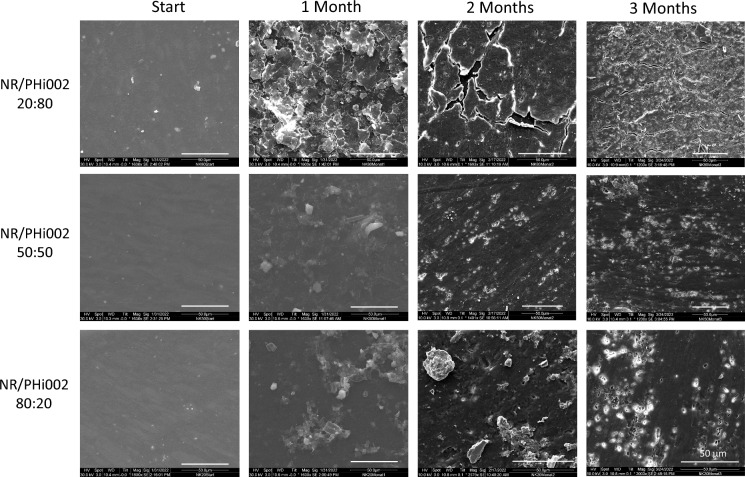
Representative SEM micrographs of NR/PHi002 blends at ratios of 20:80, 50:50, and 80:20 after 1–3 months of incubation in estuarine mud. Images were captured at 1600 × magnification. White scale bar: 50 µm

The environmental degradability of NR/PHBV composite materials was evaluated under estuarine conditions. Scanning electron microscopy revealed progressive surface alterations in all tested blends over the incubation period (Fig. 5). Initially, all samples displayed smooth and uniform surfaces. After one month, surfaces became increasingly rough and irregular, particularly in materials with higher PHBV content.

NR/PHi002 20:80 (20% NR, 80% PHBV) exhibited pronounced porosity and brittleness, with the outer layer showing extensive cracking and pore formation. In contrast, NR/PHi002 80:20 (80% NR, 20% PHBV) displayed only minor surface roughening without visible fractures. The extent of surface erosion and fragmentation correlated with the PHBV fraction.

The weight loss was 2.3 ± 0.3 mg for NR/Phi002 20:80 (corresponding to 1.8 ± 0.1%), 1.5 ± 0.1 mg for NR/PHi002 50:50 (1.5 ± 0.1%), and 1.5 ± 0.1 mg for NR/PHi002 80:20 (1.4 ± 0.1%). After rinsing, drying, and preparation of the samples for SEM imaging, no clear adhering microbial cover or biofilm was observed on the bar surfaces.

**Fig. 6:**
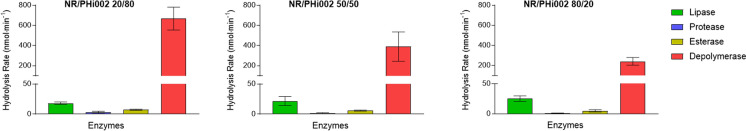
Hydrolytic degradation of the three NR/PHi002 blends by lipase, esterase, protease, and *Pl*DP in seawater, measured by pH–Stat titration at 30 °C (means ± SD, n = 3). The y-axis is discontinuous to better resolve low hydrolysis rates between 0 and 50 nmol·min^−1^

All NR-PHBV blends showed low hydrolysis rates in seawater when incubated with commercial lipase, protease, or esterase (Fig. 6). Of the commercial enzymes, lipase showed the highest hydrolysis rates ranging from 18.1 ± 2.0 nmol·min^−1^ for NR/PHi002 80:20 to 25.1 ± 4.7 nmol·min^−1^ for NR/PHi002 20:80. All NR-PHBV blends showed high hydrolysis rates when incubated with *Pl*DP, which strongly decreased with increasing natural rubber content in the blend (668.2 ± 113.4 nmol·min^−1^ for NR/PHi002 20:80, 390.4 ± 145.5 nmol·min^−1^ for NR/PHi002 50:50, and 241.2 ± 38.5 nmol·min^−1^ for NR/PHi002 80:20).

## Discussion

The biodegradability of bio-based plastics is an important aspect of their potential as sustainable alternatives to petrochemical polymers. Biodegradation proceeds in distinct stages (Pathak and Navneet 2017; Gu et al. 2024): microorganisms first colonize the polymer surface and secrete extracellular enzymes that cleave the material into shorter fragments. These oligomers, dimers, and monomers are then assimilated and metabolized as carbon and energy sources. Final mineralization occurs intracellularly under aerobic or anaerobic conditions, depending on the environment.

In this study, we systematically evaluated the enzymatic degradation of PHBV, NR, and NR/PHBV blends, revealing that biodegradation is influenced by both the polymer composition and the properties and activities of the enzymes involved. PHA depolymerases are compiled and classified in the Depolymerase Engineering Database (DED, http://www.ded.uni-stuttgart.de), established in 2009. The data base contains 587 sequence entries grouped into 8 superfamilies and 38 homologous families based on sequence similarity (Knoll et al. 2009).

Using recombinant, purified depolymerases, we modeled the initial biodeterioration phase at the laboratory scale, which is characterized by enzymatic surface attack and serves as a key determinant for subsequent degradation (Tokiwa et al. 2009). Surface morphology emerged as a particularly important factor. Biodegradation of the material begins with enzyme-driven depolymerization that occurs naturally on the surface. Because there is no mechanical action on the material by agitation or biotic activity, which might have contributed to surface damage and erosion, this leaves enzymatic action as sole driver of the observed disintegration. Comparison of PHi001 and PHi002 cast films showed that materials containing PBAT as a copolymer were more accessible to enzymatic attack, thereby enhancing biodegradation. This observation is consistent with previous studies reporting faster degradation of copolymers compared to homopolymers (Sridewi et al. 2006) and highlighting the susceptibility of the aliphatic PBAT component to enzymatic hydrolysis (Wallace et al. 2017). More broadly, these findings align with the established inverse correlation between polymer crystallinity and biodegradation rate (Sridewi et al. 2006; Wallace et al. 2017).

Complementing sequence-based classification, the BRENDA enzyme database provides a functional perspective, currently distinguishing two main types of PHA depolymerases: poly(3-hydroxybutyrate) depolymerases (3-PHB depolymerases, EC 3.1.1.75) and poly(3-hydroxyoctanoate) depolymerases (3-PHO depolymerases, EC 3.1.1.76). Their catalytic activities are substrate-specific, reflecting adaptation to the chemical structures of different PHA polymers. While 3-PHB depolymerase degrades short chain length (scl)-PHA, 3-PHO depolymerase activity is limited to medium chain length (mcl)-PHA.

Our results also show that PHB depolymerases differ markedly in activity, thermal stability, and pH stability, all of which influence their efficiency on complex polymer blends. Among the enzymes tested, *Pl*DP consistently outperformed *As*DP and *Rp*DP on both model substrates (pNPB) and commercial PHA films, likely reflecting its higher catalytic activity and better access to amorphous PHBV regions. Thermal inactivation experiments revealed that *Pl*DP and *Rp*DP lost activity rapidly above 45 °C, while *As*DP remained stable, highlighting the need to match enzyme choice to environmental and industrial conditions. Notably, some previously reported PHB depolymerases have been shown to retain activity above 50 °C (Shivakumar et al. 2011; Shivakumar 2013). Those studies employed enzymes from the fungi *Penicillium citrinum* S2 and *Fusarium solani*, suggesting fundamental functional differences between pro- and eukaryotic depolymerases.

SEM analyses confirmed that depolymerase-mediated hydrolysis of NR/PHBV blends occurs via surface erosion, characterized by progressive roughening, pore formation, and fragmentation. These morphological changes align with previous reports on PHBV hydrolysis and support the idea that increasing surface area enhances enzymatic accessibility and, consequently, the apparent activity (Koller et al. 2025). Applying a two-step enzymatic approach—pre-treatment with *Pl*DP followed by Lcp-mediated oxidative cleavage of NR—improved enzymatic degradation, as indirectly measured by oxygen consumption, likely by exposing NR surfaces previously masked by PHBV. This strategy addresses a key limitation of NR biodegradation, namely its inherent resistance due to the recalcitrant poly(cis-1,4-isoprene) backbone and reliance on oxidative conditions (Prakash et al. 2024). Primarily, the steric hindrance, which is limiting the enzyme accessibility, and the diffusion limitations of the molecular oxygen, are concurrently reduced. Due to feasible cleavage, terminal functional groups with oxygen moieties arise along the NR polymer chain and significantly reduce the natural hydrophobicity of the material. These cooperative effects lead to continuous increase in open space within the material, allowing the optimal enzyme–substrate alignment, reducing the inherent hydrophobic repulsion and ensuring a sufficient supply of molecular oxygen for NR biodegradation.

To date, five types of extracellular enzymes have been reported to degrade NR: rubber oxygenase A (roxA), rubber oxygenase B (roxB), latex clearing protein (lcp), laccase, and manganese peroxidase (Rose et al. 2005; Nayanashree and Thippeswamy 2015; Chittella et al. 2021; Andler et al. 2022; Guajardo and Andler 2024). These enzymes have been mainly characterized in *Xanthomonas* sp. 35Y and *Streptomyces* sp. K30 (Guajardo and Andler 2024). RoxA, RoxB, and Lcp catalyze the direct oxidative cleavage of poly(cis-1,4-isoprene) double bonds, yielding oligo(1,4-isoprenoids) as main products (Rose et al. 2005; Guajardo and Andler 2024). In contrast, cleavage mediated by laccases, lipoxygenases, or peroxidases depends on the presence of small redox mediators that initiate free-radical chain reactions (Rose and Steinbüchel 2005; Schulte et al. 2008). While oxygenase-catalyzed cleavage is slower than hydrolytic reactions, pre-exposing NR via PHBV removal substantially increased enzyme accessibility and activity, as confirmed by accelerated NR oxidation in our assays. This might be caused by a dramatic increase of the accessible surface after pre-conditioning of the investigated specimen.

Environmental biodegradation experiments in estuarine mud provided complementary insights into NR/PHBV behavior under natural conditions. Exposure to natural mud simulates slow degradation under environmentally relevant conditions at a low temperature (10 °C), with plastic samples buried in the sediment and subjected to naturally occurring, low enzymatic activities. SEM imaging revealed substantial surface erosion and pore formation in PHBV-rich blends within three months, whereas NR-rich blends remained largely intact. These observations highlight the recalcitrance of NR to microbial consortia in estuarine environments, likely reflecting a combination of oxygen limitation, reduced oxidative enzyme activity, and the absence of specific NR-degrading microorganisms. The pH–Stat titration was carried out to prove enzymatic cleavage. A potential mechanical degradation due to friction can be excluded by considering the enzyme-free blank approach. Measurements of the hydrolysis of the NR/PHBV blends revealed that depolymerase efficiently degrades the PHBV fraction in seawater with the depolymerase activity decreasing progressively with increasing NR content in the blends. In contrast, lipase, protease, and esterase have a low potential for degrading PHBV and NR, as evidenced by consistently low hydrolysis rates across all blend compositions. Depolymerase-producing microorganisms are known from a wide range of marine environments (Suzuki et al. 2021). However, their abundance and enzymatic activity within these habitats are largely unknown. While *in-vitro* measurements indicate a high degradability, decomposition experiments in mud reveal a rather slow degradation. This discrepancy suggests either a limited depolymerase activity within the mud, the presence of less efficient depolymerases variants, or a generally low abundance of depolymerase-producing microorganisms. Overall, the results underscore that biodegradability depends not only on polymer composition but also on the environmental conditions. This comprises enzyme availability and favorable physico-chemical conditions, including humidity and temperature (Miksch et al. 2022).

In summary, our study systematically evaluated the biodegradability of NR, PHBV, and NR/PHBV blends, showing that degradation is shaped by polymer composition, surface properties, and environmental context. PHBV is readily hydrolyzed under laboratory and natural conditions, whereas NR is more resistant, highlighting the need to balance mechanical performance with environmental degradability when designing multi-component composites. Factors such as crystallinity and surface morphology strongly influence accessibility to microbial or enzymatic attack. Overall, these findings emphasize that biodegradability emerges from the interplay between material structure and environmental conditions, providing guiding principles for the development of mechanically robust, environmentally compatible bioplastics to support circular economy objectives.

## Data Availability

Original data can be provided upon reasonable request.

## References

[CR1] Amor SR, Rayment T, Sanders JKM (1991) Poly(hydroxybutyrate) *in vivo*: NMR and x-ray characterization of the elastomeric state. Macromolecules 24:4583–4588. 10.1021/ma00016a017

[CR2] Andler R, Guajardo C, Sepúlveda C et al (2022) Biodegradation of rubber in cultures of *Rhodococcus rhodochrous* and by its enzyme latex clearing protein. Biodegradation 33:609–620. 10.1007/s10532-022-09998-7 36197531 10.1007/s10532-022-09998-7

[CR3] Bhatt R, Shah D, Patel KC, Trivedi U (2008) PHA–rubber blends: synthesis, characterization and biodegradation. Bioresour Technol 99:4615–4620. 10.1016/J.BIORTECH.2007.06.054 17764931 10.1016/j.biortech.2007.06.054

[CR4] Bode HB, Zeeck A, Plückhahn K, Jendrossek D (2000) Physiological and chemical investigations into microbial degradation of synthetic poly(cis-1,4-isoprene). Appl Environ Microbiol 66:3680–3685. 10.1128/AEM.66.9.3680-3685.2000 10966376 10.1128/aem.66.9.3680-3685.2000 PMC92206

[CR5] Bradford M (1976) A rapid and sensitive method for the quantitation of microgram quantities of protein utilizing the principle of protein-dye binding. Anal Biochem 72:248–254. 10.1016/0003-2697(76)90527-3 942051 10.1016/0003-2697(76)90527-3

[CR6] Cheng Y, Wei Y, Wu H et al (2024) Biodegradation of vulcanized natural rubber by enriched bacterial consortia. Chem Eng J 481:148685. 10.1016/j.cej.2024.148685

[CR7] Chittella H, Yoon LW, Ramarad S, Lai Z-W (2021) Rubber waste management: a review on methods, mechanism, and prospects. Polym Degrad Stab 194:109761. 10.1016/j.polymdegradstab.2021.109761

[CR8] de Koning GJM, Lemstra PJ (1992) The amorphous state of bacterial poly[(R)-3-hydroxyalkanoate]in vivo. Polymer 33:3292–3294. 10.1016/0032-3861(92)90249-V

[CR9] Efremenko E, Stepanov N, Senko O et al (2024) Using fungi in artificial microbial consortia to solve bioremediation problems. Microorganisms 12:531. 10.3390/microorganisms12030470 38543521 10.3390/microorganisms12030470 PMC10974216

[CR10] Frank C, Emmerstorfer-Augustin A, Rath T et al (2023) Bio-polyester/rubber compounds: fabrication, characterization, and biodegradation. Polymers (Basel). 10.3390/POLYM15122593 37376240 10.3390/polym15122593 PMC10304462

[CR11] Gibson DG, Young L, Chuang R-Y et al (2009) Enzymatic assembly of DNA molecules up to several hundred kilobases. Nat Methods 6:343–345. 10.1038/nmeth.1318 19363495 10.1038/nmeth.1318

[CR12] Gu J-D, Ford T, Mitton B, Mitchell R (2024) Research on biodeteriotration of plastics. Int Biodeterior Biodegrad 186:105699. 10.1016/j.ibiod.2023.105699

[CR13] Guajardo C, Andler R (2024) Challenges and perspectives in enzymatic polymer fragmentation: the case of rubber and polyethylene terephthalate. J Clean Prod 450:141875. 10.1016/j.jclepro.2024.141875

[CR14] Hahn T, Alzate MO, Leonhardt S et al (2024) Current trends in medium‐chain‐length polyhydroxyalkanoates: microbial production, purification, and characterization. Eng Life Sci 24:2300211. 10.1002/ELSC.202300211 38845815 10.1002/elsc.202300211 PMC11151071

[CR15] Jendrossek D, Handrick R (2002) Microbial degradation of polyhydroxyalkanoates. Annu Rev Microbiol 56:403–432. 10.1146/annurev.micro.56.012302.160838 12213937 10.1146/annurev.micro.56.012302.160838

[CR16] Jo M, Jang Y, Lee E et al (2022) The modification of poly(3-hydroxybutyrate-co-4-hydroxybutyrate) by melt blending. Polymers (Basel) 14:1725. 10.3390/POLYM14091725 35566895 10.3390/polym14091725 PMC9106057

[CR17] Knoll M, Hamm TM, Wagner F et al (2009) The PHA depolymerase engineering database: a systematic analysis tool for the diverse family of polyhydroxyalkanoate (PHA) depolymerases. BMC Bioinformatics 10:89. 10.1186/1471-2105-10-89 19296857 10.1186/1471-2105-10-89 PMC2666664

[CR18] Koike T, Muranaka Y, Maki T (2024) Kinetic analysis of the enzymatic degradation behavior of polyhydroxyalkanoate (PHA) based on its solid-state structure. React Funct Polym 202:105950. 10.1016/j.reactfunctpolym.2024.105950

[CR19] Koller M, Heeney D, Mukherjee A (2025) Biodegradability of polyhydroxyalkanoate (PHA) biopolyesters in nature: a review. Biodegradation 36:1–65. 10.1007/S10532-025-10164-Y/FIGURES/3 10.1007/s10532-025-10164-y PMC12339601 40788578

[CR01] Lin-Cereghino J, Wong WW, Xiong S, Giang W, Luong LT, Vu J, Johnson SD, Lin-Cereghino GP (2005) Condensed protocol for competent cell preparation and transformation of the methylotrophic yeast Pichia pastoris. Biotechniques 38(1):44, 46, 48. 10.2144/05381BM04 10.2144/05381BM04 PMC2504082 15679083

[CR20] Lyshtva P, Voronova V, Barbir J et al (2024) Degradation of a poly(3-hydroxybutyrate-co-3-hydroxyvalerate) (PHBV) compound in different environments. Heliyon 10:e24770. 10.1016/J.HELIYON.2024.E24770 38322905 10.1016/j.heliyon.2024.e24770 PMC10844030

[CR21] Marchessault RH, Yu G (2002) Crystallization and material properties of polyhydroxyalkanoates PHAs. Biopolymers Online. 10.1002/3527600035.BPOL3B07

[CR22] Miksch L, Köck M, Gutow L, Saborowski R (2022) Bioplastics in the sea: rapid *in-vitro* evaluation of degradability and persistence at natural temperatures. Front Mar Sci 9:920293. 10.3389/FMARS.2022.920293/BIBTEX

[CR23] Näätsaari L, Mistlberger B, Ruth C et al (2012) Deletion of the *Pichia pastoris KU70* homologue facilitates platform strain generation for gene expression and synthetic biology. PLoS ONE 7:e39720. 10.1371/journal.pone.0039720 22768112 10.1371/journal.pone.0039720 PMC3387205

[CR24] Nachtnebel M, Rastel M, Mayrhofer C et al (2017) The fracture behavior of particle modified polypropylene – 3D reconstructions and interparticle distances. Polymer 126:65–73. 10.1016/j.polymer.2017.08.029

[CR25] Nayanashree G, Thippeswamy B (2015) Biodegradation of natural rubber by laccase and manganese peroxidase enzyme of *Bacillus subtilis*. Environ Process 2:761–772. 10.1007/s40710-015-0118-y

[CR26] Nguyen LH, Nguyen HD, Tran PT et al (2020) Biodegradation of natural rubber and deproteinized natural rubber by enrichment bacterial consortia. Biodegradation 31:303–317. 10.1007/s10532-020-09911-0 32914250 10.1007/s10532-020-09911-0

[CR27] Pandey A, Adama N, Adjallé K, Blais JF (2022) Sustainable applications of polyhydroxyalkanoates in various fields: a critical review. Int J Biol Macromol 221:1184–1201. 10.1016/j.ijbiomac.2022.09.098 36113591 10.1016/j.ijbiomac.2022.09.098

[CR28] Pathak VM, Navneet (2017) Review on the current status of polymer degradation: a microbial approach. Bioresour Bioprocess 4:15. 10.1186/s40643-017-0145-9

[CR29] Prakash T, Yadav SR, Bürger M, Jendrossek D (2024) Cleavage of natural rubber by rubber oxygenases in Gram-negative bacteria. Appl Microbiol Biotechnol 108:1–9. 10.1007/S00253-023-12940-3/FIGURES/2 38305904 10.1007/s00253-023-12940-3 PMC10837239

[CR30] Ranakoti L, Gangil B, Mishra SK et al (2022) Critical review on polylactic acid: properties, structure, processing, biocomposites, and nanocomposites. Materials 15(12):4312. 10.3390/MA15124312 35744371 10.3390/ma15124312 PMC9228835

[CR31] Roberts C, Edwards S, Vague M et al (2020) Environmental consortium containing *Pseudomonas* and *Bacillus* species synergistically degrades polyethylene terephthalate plastic. mSphere. 10.1128/msphere.01151-20 33361127 10.1128/mSphere.01151-20 PMC7763552

[CR32] Rose K, Steinbüchel A (2005) Biodegradation of natural rubber and related compounds: recent insights into a hardly understood catabolic capability of microorganisms. Appl Environ Microbiol 71:2803–2812. 10.1128/AEM.71.6.2803-2812.2005 15932971 10.1128/AEM.71.6.2803-2812.2005 PMC1151847

[CR33] Rose K, Tenberge KB, Steinbüchel A (2005) Identification and characterization of genes from *Streptomyces* sp. strain K30 responsible for clear zone formation on natural rubber latex and poly(cis-1,4-isoprene) rubber degradation. Biomacromol 6:180–188. 10.1021/bm0496110 10.1021/bm0496110 15638519

[CR34] Salinas J, Carpena V, Martínez-Gallardo MR et al (2023) Development of plastic-degrading microbial consortia by induced selection in microcosms. Front Microbiol. 10.3389/fmicb.2023.1143769 37113240 10.3389/fmicb.2023.1143769 PMC10126402

[CR35] Salinas J, Martínez-Gallardo MR, Jurado MM et al (2024) Microbial consortia for multi-plastic waste biodegradation: selection and validation. Environ Technol Innov 36:103887. 10.1016/j.eti.2024.103887

[CR36] Schulte C, Arenskötter M, Berekaa MM et al (2008) Possible involvement of an extracellular superoxide dismutase (SodA) as a radical scavenger in poly(cis-1,4-Isoprene) degradation. Appl Environ Microbiol 74:7643–7653. 10.1128/AEM.01490-08 18952871 10.1128/AEM.01490-08 PMC2607186

[CR37] Shivakumar S (2013) Poly- β -hydroxybutyrate (PHB) depolymerase from *Fusarium solani* Thom. J Chem. 10.1155/2013/406386

[CR38] Shivakumar S, Jagadish SJ, Zatakia H, Dutta J (2011) Purification, characterization and kinetic studies of a novel poly(β) hydroxybutyrate (PHB) depolymerase PhaZPen from *Penicillium citrinum* S2. Appl Biochem Biotechnol 164:1225–1236. 10.1007/s12010-011-9208-0 21369777 10.1007/s12010-011-9208-0

[CR39] Skariyachan S, Manjunatha V, Sultana S et al (2016) Novel bacterial consortia isolated from plastic garbage processing areas demonstrated enhanced degradation for low density polyethylene. Environ Sci Pollut Res 23:18307–18319. 10.1007/s11356-016-7000-y 10.1007/s11356-016-7000-y 27278068

[CR40] Sridewi N, Bhubalan K, Sudesh K (2006) Degradation of commercially important polyhydroxyalkanoates in tropical mangrove ecosystem. Polym Degrad Stab 91:2931–2940. 10.1016/j.polymdegradstab.2006.08.027

[CR41] Suzuki M, Tachibana Y, Kasuya K (2021) Biodegradability of poly(3-hydroxyalkanoate) and poly(ε-caprolactone) via biological carbon cycles in marine environments. Polym J 53:47–66. 10.1038/S41428-020-00396-5

[CR42] Tokiwa Y, Calabia BP, Ugwu CU, Aiba S (2009) Biodegradability of plastics. Int J Mol Sci 10:3722–3742. 10.3390/ijms10093722 19865515 10.3390/ijms10093722 PMC2769161

[CR43] Wallace PW, Haernvall K, Ribitsch D et al (2017) *Pp*Est is a novel PBAT degrading polyesterase identified by proteomic screening of *Pseudomonas pseudoalcaligenes*. Appl Microbiol Biotechnol 101:2291–2303. 10.1007/s00253-016-7992-8 27872998 10.1007/s00253-016-7992-8 PMC5320007

[CR44] Zankel A, Chernev B, Brandl C et al (2008) Assessment of beam damage in polymers caused by in situ ESEM analysis using IR spectroscopy. Macromol Symp 265:156–165. 10.1002/masy.200850517

[CR45] Zeghal E, Vaksmaa A, Vielfaure H et al (2021) The potential role of marine fungi in plastic degradation—a review. Front Mar Sci 8:205–223. 10.3389/fmars.2021.738877

[CR46] Zhao X, Ji K, Kurt K, Cornish K, Vodovotz Y (2019) Optimal mechanical properties of biodegradable natural rubber-toughened PHBV bioplastics intended for food packaging applications. Food Packag Shelf Life 21:100348. 10.1016/J.FPSL.2019.100348

